# Effect of clonal testing on the efficiency of genomic evaluation in forest tree breeding

**DOI:** 10.1038/s41598-022-06952-8

**Published:** 2022-02-22

**Authors:** J. Stejskal, J. Klápště, J. Čepl, Y. A. El-Kassaby, M. Lstibůrek

**Affiliations:** 1 Faculty of Forestry and Wood Sciences, Czech University of Life Sciences, Kamýcká 1176, 165 21 Praha, Czech Republic; 2 Scion (New Zealand Forest Research Institute Ltd.), 49 Sala Street, Whakarewarewa, 3010 Rotorua, New Zealand; 3 Department of Forest and Conservation Sciences, Faculty of Forestry, University of British Columbia, Vancouver, BC V6T 1Z4 Canada

**Keywords:** Genomics, Plant breeding

## Abstract

Through stochastic simulations, accuracies of breeding values and response to selection were assessed under traditional pedigree-(BLUP) and genomic-based evaluation methods (GBLUP) in forest tree breeding. The latter provides a methodological foundation for genomic selection. We evaluated the impact of clonal replication in progeny testing on the response to selection realized in seed orchards under variable marker density and target effective population sizes. We found that clonal replication in progeny trials boosted selection accuracy, thus providing additional genetic gains under BLUP. While a similar trend was observed for GBLUP, however, the added gains did not surpass those under BLUP. Therefore, breeding programs deploying extensive progeny testing with clonal propagation might not benefit from the deployment of genomic information. These findings could be helpful in the context of operational breeding programs.

## Introduction

Genomic selection (GS) has garnered increased attention for its potential to deliver significant genetic gains per unit of time and cost. In traditional genetic evaluation, phenotypic performance is regressed on the identity-by-descent (IBD) probabilities inferred from pedigrees, producing the best linear unbiased predictions (BLUP) of additive genetic (breeding) values. However, when genetic covariance is estimated from DNA markers (e.g., SNP), genomic-based predictions (GBLUP) of true breeding values may provide additional benefits over that of the traditional BLUP, mainly when used in the context of GS.

In forest trees, recurrent GS is based on establishing training populations (i.e., marker-trait associations). These, in turn, eliminate, to a certain extent (loss of accuracy over time)**,** the needed phenotypic evaluation phase of selection candidates. Using computer simulation, the theoretical potential of GS in tree breeding was demonstrated with the conclusion that it could radically increase breeding efficiency^1,2^. They assessed GS scenarios under variable narrow-sense heritability (*h*^2^), number of QTLs, marker density, and effective population size (*N*_*e*_). Recent studies utilizing empirical data reported additional prediction accuracies^3,4^.

Unlike the studies mentioned above, the genetic response to selection was recently simulated in a seed production population, providing a more realistic basis for comparing actual genetic gains available in forest reproductive material^5^. In particular, they expressed the efficiency of GBLUP/BLUP protocols based on the respective ranking of selection candidates and genetic gains provided in forest reproductive material. Furthermore, a combination of low *h*^2^, high *N*_*e*_, and dense marker coverage resulted in the maximum genomic prediction efficiency and added within-family selection accuracy (exploitation of the Mendelian sampling term). Adoption of GS in operational breeding programs is challenging in predominantly outbred forest tree species. These are characterized by: (1) long generation intervals, (2) sensitivity to inbreeding, i.e., breeding relying on high *N*_*e*_, (3) fast gametic-phase disequilibrium decay, (4) considerable temporal and spatial environmental sensitivity, and (5) large genome sizes (especially in conifers), requiring dense SNP genotyping involving many individuals^6^. Regarding forest tree species' spatial and temporal sensitivity, excessive field experiments are required to assess genotype-by-environment interactions and age-age correlations on top of complicated covariance structure among multiple traits and its shift with selection. These issues are specific to populations, traits, ages, and environmental conditions and require testing hundreds of families in several environments over an excessive time scale^7^.

Genetically improved seed production predominantly relies on seed orchards, i.e., bulk seed from open-pollinated crosses, capturing additive genetic variance. Additionally, one can exploit non-additive genetic effects through the mass deployment of full-sib families (dominance) or clonal mixtures (dominance + epistasis). Apart from deploying improved forest reproductive material, one can clonally replicate selection candidates (offspring genotypes) in progeny trials, enhancing the precision of forward selection^8,9^. In Sweden, clonal replication in progeny testing has provided operational benefits in the Norway spruce breeding program by boosting the within-family response to selection while minimizing genetic diversity loss^9,10^. Under fixed progeny test size, a trade-off exists between the family size and the number of clonal propagules per genotype (*N*_*R*_), i.e., clonal size^11^.

Here, building on our earlier stochastic simulations^5^, we evaluated the impact of clonal replication in progeny testing on the efficiency of BLUP and GBLUP evaluation and the actual genetic response realized in seed orchards. Specifically, we assessed the combined effect of marker density, effective population size (*N*_*e*_), family size, and *N*_*R*_.

## Methods

We utilized a stochastic simulation model developed in R^12^. We created parental and offspring populations^5^ using the function “glSim” implemented within the R package “adegenet”^13^ to generate allelic frequencies in a founder population. Linkage disequilibrium (*LD*) was set to reflect typical values in outcrossed forest trees^6^. We generated offspring populations (50 parents) of two different sizes using a single pair mating design (SPM) to evaluate the impact of family size (80/160), so the overall population size varied from 2050 to 4050 individuals.

Bi-allelic marker data were simulated for the full-sib families using the function “genomesim” implemented within the R package “pedantics”^14^. We set the number of markers (SNPs) per centiMorgan (cM) to 1, 5, and 10 covering chromosome lengths of 120 cM. In total, two chromosomes (linkage groups) were simulated, comparably to the previous studies^3^, with the maximum number of markers equal to 2400. As the impact of traits' genetic architecture was evaluated earlier^5^, we modeled only a fixed QTL number (*N*_*QTL*_ = 200). QTL effects were randomly assigned to selected loci and were sampled from a standardized normal distribution to emulate polygenic traits. We generated phenotypic data as the sum of allelic effects across all QTL loci with the addition of residual effects reflecting *h*^2^ = 0.2, which approximates growth traits in forest tree species. Clonal replicates were derived as the sum of a genotypic value and the average of *N*_*R*_ independent samples of residual effects. We conducted 200 independent stochastic iterations of the above scenarios.

We conducted separate genetic evaluations in ASReml software V.3^15^ for pedigree- (BLUP) and genomic-based (GBLUP) relationships to predict offspring breeding value (BV; i.e., forward selection) using the animal model in the REML framework^16^. The marker-based relationship matrix ***G*** was constructed as follows^17^:

$${\varvec{G}}=\frac{{\varvec{Z}}{\varvec{Z}}^{\prime}}{2\sum {\varvec{p}}\left(1-{\varvec{p}}\right)}$$

where ***Z*** is ***M***—***P***, ***M*** is the marker matrix containing genotypes coded as 0, 1, and 2 for the first allele homozygote, heterozygote, and second allele homozygote, and ***P*** is the vector of doubled frequencies of the second allele, ***p*** is the frequency of the second allele at the loci.

Breeding value (BV) accuracy was calculated as the correlation between their predicted (genetic evaluation of both BLUP and GBLUP strategies) and true values (as determined by the sum of simulated allelic effects). Next, the reported standard error of the overall accuracy across 200 iterations (calculated as the respective standard deviation divided by the square root of the iteration count). Following the genetic evaluation, a set of unrelated offspring with top breeding values (considered as parents in seed orchards) was chosen by mathematical programming^18^ to meet the predetermined effective population size (*N*_*e*_ = 5, 10, 20, and 25), thus maximizing the genetic response^19^. The method selects the best set of offspring individuals, maximizing the average additive genetic value (genetic gain) while meeting the declared effective population size (constraint). Relatedness among the selected trees was not permitted to avoid inbreeding in the seed orchard's crop. Optimization was conducted in Gurobi software ^18^. Details on the optimization algorithm are provided in Lstibůrek and Hodge ^19^.

## Results

### Accuracy of predicted breeding values

Table 1 provides BV's accuracies at variable family size, *N*_*R*_, and marker density. Under GBLUP, a steady increase in BV's accuracy is visible with higher marker density (mainly between 1 and 5 SNPs/cM). BV's accuracy under GBLUP was greater than BLUP under all investigated scenarios after marker density reached 5 SNPs/cM. However, under 1 SNP/cM, BV's accuracies of GBLUP were inferior to BLUP irrespective of *N*_*R*_ and the family size.

**Table 1:** Accuracy of breeding values as a function of family size, clonal size *N*_*R*_, and marker density (SNPs/cM).

	Family size (80)						Family size (160)					
	*N*_*R*_ = 1		*N*_*R*_ = 6		*N*_*R*_ = 12		*N*_*R*_ = 1		*N*_*R*_ = 6		*N*_*R*_ = 12	
	BLUP	GBLUP	BLUP	GBLUP	BLUP	GBLUP	BLUP	GBLUP	BLUP	GBLUP	BLUP	GBLUP
1 SNP/cM	0.700 (0.003)	0.501 (0.002)	0.830 (0.002)	0.545 (0.001)	0.890 (0.001)	0.551 (0.004)	0.718 (0.003)	0.528 (0.004)	0.832 (0.002)	0.546 (0.003)	0.887 (0.002)	0.549 (0.003)
5 SNPs/cM	0.787 (0.002)		0.906 (0.001)		0.932 (0.001)		0.837 (0.002)		0.926 (0.001)		0.945 (0.001)	
10 SNPs/cM	0.803 (0.002)		0.920 (0.001)		0.945 (0.001)		0.850 (0.002)		0.939 (0.001)		0.957 (0.001)	

*N*_*R*_ = 1 means no-cloning (1 ramet per clone).

Clonal replication boosted accuracies of both BLUP and GBLUP evaluations across all marker densities and family sizes. The difference is visible primarily between one to six clonal copies, while additional clonal replications (up to 12) provided lower increments. At low marker density (1 SNP/cM), the accuracy of GBLUP ranged from 62 to 74% of that under BLUP at both family sizes. Assuming the family size 80, clonal replication reduced relative accuracy of GBLUP over BLUP, e.g., reduction from 72% (*N*_*R*_ = 1) to 66% (*N*_*R*_ = 6) to 62% (*N*_*R*_ = 12). The same observation was made under 5 SNPs/cM, namely the reduction from 112% (*N*_*R*_ = 1) to 109% (*N*_*R*_ = 6) to 105% (*N*_*R*_ = 12). Similarly, under 10 SNPs/cM, relative accuracies dropped from 115% (*N*_*R*_ = 1) to 111% (*N*_*R*_ = 6) to 106% (*N*_*R*_ = 12). A similar trend was observed under family size 160. As expected, absolute accuracies were boosted by family size under both BLUP and GBLUP except for the lowest marker coverage.

### Selection response

In Table 2, we present differences in the standardized response to selection between GBLUP and BLUP. Relative differences are provided in Table S2. The impact of added marker density was most significant between 1 and 5 SNPs/cM, and it was diminishing towards the 10 SNPs/cM, primarily under the family size 80.

**Table 2:** Differences in standardized genetic gains between GBLUP and BLUP for combinations of *N*_*R*_, *N*_*e*_ (5, 10, 20, 25), marker density (1, 5, 10 SNPs/cM), and family size 80 (top table) and 160 (bottom table).

Family size = 80	*N*_*R*_ (1, 6, 12)											
	1				6				12			
	*N*_*e*_ = 5	*N*_*e*_ = 10	*N*_*e*_ = 20	*N*_*e*_ = 25	*N*_*e*_ = 5	*N*_*e*_ = 10	*N*_*e*_ = 20	*N*_*e*_ = 25	*N*_*e*_ = 5	*N*_*e*_ = 10	*N*_*e*_ = 20	*N*_*e*_ = 25
1 SNP/cM	− 0.52*	− 0.49*	− 0.41*	− 0.38	− 0.97*	− 0.92*	− 0.84*	− 0.80*	− 1.15*	− 1.09*	− 1.01*	− 0.96*
5 SNPs/cM	0.36	0.33	0.31	0.29	0.21	0.21	0.19	0.18	0.09	0.11	0.10	0.09
10 SNPs/cM	0.41*	0.38*	0.36*	0.35	0.3	0.29	0.26	0.25	0.22	0.22	0.19	0.18

Family size = 160	*N*_*R*_ (1, 6, 12)											
	1				6				12			
	*N*_*e*_ = 5	*N*_*e*_ = 10	*N*_*e*_ = 20	*N*_*e*_ = 25	*N*_*e*_ = 5	*N*_*e*_ = 10	*N*_*e*_ = 20	*N*_*e*_ = 25	*N*_*e*_ = 5	*N*_*e*_ = 10	*N*_*e*_ = 20	*N*_*e*_ = 25
1 SNP/cM	− 0.53*	− 0.51*	− 0.44*	− 0.41*	− 1.1*	− 1.05*	− 0.94*	− 0.90*	− 1.29*	− 1.23*	− 1.13*	− 1.07*
5 SNPs/cM	0.52*	0.48*	0.45*	0.43*	0.28	0.28	0.26	0.25	0.17	0.17	0.15	0.15
10 SNPs/cM	0.58*	0.54*	0.49*	0.49*	0.33	0.34	0.31	0.31	0.25	0.23	0.22	0.21

An asterisk indicates significant differences (alpha = 0.05). *N*_*R*_ = 1 means no-cloning (1 ramet per clone).

Under clonal replication (*N*_*R*_ = 6–12), GBLUP yielded minor benefit in selection response under 5–10 SNPs/cM, but the difference was not statistically significant (alpha = 0.05). The impact of *N*_*R*_ on the absolute selection response of both methods is prominent irrespective of the marker density, primarily between 1 and 6 clonal replicates (yet additional gain was generated under *N*_*R*_ = 12). While the clonal replication improves gains of both evaluation methods, the major boost of selection response was observed under the BLUP. Under the lowest marker density, i.e., 1 SNP/cM, BLUP generated a higher selection response in the range of app. 0.4–1.3 standard deviations. Under *N*_*R*_ = 1 (no cloning), GBLUP was superior to BLUP, primarily under higher marker densities and larger families (see Table 2, *N*_*R*_ = 1). The above trends were generally true across the range of *N*_*e*_, yet the added difference between the two methods was diluted at larger *N*_*R*_. Under *N*_*R*_ = 1 and moderate marker density (5 SNP/cM), a larger family size (160) boosted the difference between the two methods. Note that values in Table 2 are differences among standardized genetic gains of BLUP and GBLUP. Thus, they are not reflecting baseline genetic gains, e.g., a significant drop of selection response with added *N*_*e*_. Under *N*_*R*_ = 1, low marker density (1 SNP/cM), *N*_*e*_ = 5, the absolute difference − 0.52 (Table 2) reflects standardized gains of 0.88 (GBLUP) and 1.4 (BLUP). Assuming the same parameters, but *N*_*e*_ = 25, the absolute difference − 0.38 reflects a significant drop in standardized gains due to lower selection intensity, i.e., 0.13 (GBLUP) and 0.51 (BLUP). For clarity, standardized genetic gains of all strategies are provided in Table S1. Relative genetic gains, i.e., ratios of the standardized genetic response of GBLUP/BLUP, are provided in Table S2.

## Discussion

Here, we estimated the relative efficiency of the genomic evaluation protocol over the traditional phenotypic alternative. Our findings resemble animal and plant breeding studies, i.e., the added prediction accuracy and anticipated selection response under genomic evaluation. This relative superiority of GBLUP is conditional on dense marker coverage, lower narrow-sense heritabilities, and the presence of the population-wise linkage disequilibrium^1,3,5,20,21^. In operational tree breeding programs, additional factors contribute to the breeding efficiency, e.g., sizes of breeding and production populations, mating design, progeny test size and configuration, maximum acceptable inbreeding rate, extend of genotype by environment interactions, cost and time parameters of breeding activities, etc. (see^7^ for introduction to forest tree genetics and breeding).

The novelty of our comparison is attributed to the inclusion of clonal replication in progeny test trials as used in operational tree breeding programs to boost selection accuracy. The main added value of the GBLUP evaluation is its ability to capitalize on capturing within-family additive genetic variance and unmasking cryptic relatedness^22,23^. Under all investigated scenarios, the relative genetic gain efficiency of GBLUP decreased with added clonal propagules per offspring individual (*N*_*R*_). Under 1 SNP/cM, BLUP provided a significantly higher genetic response over the GBLUP across the whole range of *N*_*e*_ and family sizes. Both evaluations yielded comparable genetic gains with denser marker coverage (5–10 SNPs/cM); differences were not significant (alpha = 0.05). This finding implies that combining both cloning and genomic evaluation does not bring added genetic response. Thus, our results could inspire breeders to consider two broader alternatives. One involves investing resources into clonal test trials, the other one to genomic evaluation. In agreement with previous studies^8,9^, relatively low *N*_*R*_ (6) provided sufficient accuracy. While both strategies benefited in prediction accuracies from the added *N*_*R*_, their relative efficiency was equalized by applying diversity constraint (*N*_*e*_) in selection. This is a clear message to operational forest tree breeding programs. Without cloning, the superiority of GBLUP is limited to low *h*^2^, large family sizes, and higher marker coverage (5–10 SNPs/cM). Genetic response in smaller families is limited due to the model oversaturation under dense marker coverage^23^. On the contrary, large family sizes (160 offspring per cross) become impractical in many species, even though they are not prone to oversaturation and provide options for higher selection intensity (larger number of selection candidates). GBLUP provided no additional benefit over the BLUP alternative across the diversity range in production populations scenarios (seed orchards) with clonal replication.

There are practical scenarios under which GBLUP could become economically more feasible. These include programs with too costly or unavailable clonal propagation technology. In analogy, SNP genotyping platform has been developed and is currently operationally feasible in a limited number of forest tree species. As forest trees are long-lived perennials with generational intervals spanning decades, the principal added benefit of genomic selection is reducing the breeding cycle's length. Therefore, GBLUP is becoming a viable platform in this context. Our conclusions are relevant to full-scale operational tree breeding programs that capture general combining ability with repeated cycles of control crosses (single-pair mating, progeny testing, and selection). Adaptive genetic response in natural populations, in theory, could be enabled by the GBLUP evaluation based on the SNP chip platform. However, this is limited by the magnitude of genetic covariance, i.e., the product of genetic coancestry and the respective genetic variance in natural populations. Future research could investigate, by stochastic simulation, the genomic-based single-step model (HBLUP) augmented by clonal replication in progeny testing.

## Data availability

In our study, we described a stochastic simulation model and compared hypothetical breeding strategies. No real-world data of any species have been used throughout the study. However, output data have been outlined and published in tables and figures included in the manuscript. The complete R code was submitted as a compressed folder within supplements (S3 file).

## Supplementary Information

Supplementary Table S1.

Supplementary Table S2.

Supplementary Information.
