# Supplementary material for: Effect of clonal testing on the efficiency of genomic evaluation in forest tree breeding
Source: Sci Rep. 2022 Feb 22;12:3033. doi: 10.1038/s41598-022-06952-8 (PMC8864020; doi:10.1038/s41598-022-06952-8)
Supplement: Supplementary file 1 — Supplementary Table S1. [file 41598_2022_6952_MOESM1_ESM.docx]

**Table S1.** Genetic gain under cloning of BLUP/GBLUP for combinations of *h^2^* = 0.2, *N_QTL_* = 200, *N_e_* (5, 10, 20, 25), and marker density (1, 5, 10 SNPs / cM). Range of clonal replications 1-12.

| Family size = 80 | *N_R_* (1, 6, 12) | | | | | | | | | | | | |
| --- | --- | --- | --- | --- | --- | --- | --- | --- | --- | --- | --- | --- | --- |
|  | 1 | | | | 6 | | | | | 12 | | | |
|  | *N_e_* = 5 | *N_e_* = 10 | *N_e_* = 20 | *N_e_* = 25 | *N_e_* = 5 | *N_e_* = 10 | *N_e_* = 20 | *N_e_* = 25 | *N_e_* = 5 | | *N_e_* = 10 | *N_e_* = 20 | *N_e_* = 25 |
| 1 SNP/cM | 1.40/0.88 | 1.14/0.65 | 0.74/0.33 | 0.51/0.13 | 1.96/0.99 | 1.65/0.73 | 1.21/0.37 | 0.97/0.17 | 2.14/0.99 | | 1.84/0.75 | 1.40/0.39 | 1.14/0.18 |
| 5 SNPs/cM | 1.56/1.92 | 1.28/1.61 | 0.88/1.19 | 0.65/0.94 | 2.12/2.33 | 1.82/2.03 | 1.38/1.57 | 1.12/1.30 | 2.35/2.44 | | 2.02/2.13 | 1.56/1.66 | 1.30/1.39 |
| 10 SNPs/cM | 1.33/1.74 | 1.06/1.44 | 0.66/1.02 | 0.42/0.77 | 1.87/2.17 | 1.56/1.85 | 1.12/1.38 | 0.88/1.13 | 2.05/2.27 | | 1.73/1.95 | 1.29/1.48 | 1.04/1.22 |
| Family size = 160 | *N_R_* (1, 6, 12) | | | | | | | | | | | | |
|  | 1 | | | | 6 | | | | | 12 | | | |
|  | *N_e_* = 5 | *N_e_* = 10 | *N_e_* = 20 | *N_e_* = 25 | *N_e_* = 5 | *N_e_* = 10 | *N_e_* = 20 | *N_e_* = 25 | *N_e_* = 5 | | *N_e_* = 10 | *N_e_* = 20 | *N_e_* = 25 |
| 1 SNP/cM | 1.43/0.90 | 1.16/0.65 | 0.74/0.30 | 0.50/0.09 | 2.02/0.92 | 1.71/0.66 | 1.25/0.31 | 1.00/0.10 | 2.22/0.93 | | 1.90/0.67 | 1.44/0.31 | 1.18/0.11 |
| 5 SNPs/cM | 1.48/2.00 | 1.21/1.69 | 0.80/1.25 | 0.56/0.99 | 2.07/2.35 | 1.75/2.03 | 1.31/1.57 | 1.05/1.30 | 2.15/2.32 | | 1.82/1.99 | 1.35/1.50 | 1.09/1.24 |
| 10 SNPs/cM | 1.48/2.06 | 1.21/1.75 | 0.82/1.31 | 0.57/1.06 | 2.11/2.44 | 1.79/2.13 | 1.35/1.66 | 1.09/1.40 | 2.30/2.55 | | 1.99/2.22 | 1.53/1.75 | 1.27/1.48 |
