# Supplementary material for: Effect of clonal testing on the efficiency of genomic evaluation in forest tree breeding
Source: Sci Rep. 2022 Feb 22;12:3033. doi: 10.1038/s41598-022-06952-8 (PMC8864020; doi:10.1038/s41598-022-06952-8)
Supplement: Supplementary file 2 — Supplementary Table S2. [file 41598_2022_6952_MOESM2_ESM.docx]

**Table S2.** Relative differences in genetic gains (ratio of standardized genetic gains of GBLUP and BLUP in %) for combinations of *N_R_*, *N_e_* (5, 10, 20, 25), marker density (1, 5, 10 SNPs / cM), and family size 80 (top table) and 160 (bottom table). Significant differences (alpha = 0.05%) are indicated by asterisk.

| Family size = 80 | *N_R_* (1, 6, 12) | | | | | | | | | | | | |
| --- | --- | --- | --- | --- | --- | --- | --- | --- | --- | --- | --- | --- | --- |
|  | 1 | | | | 6 | | | | | 12 | | | |
|  | *N_e_* = 5 | *N_e_* = 10 | *N_e_* = 20 | *N_e_* = 25 | *N_e_* = 5 | *N_e_* = 10 | *N_e_* = 20 | *N_e_* = 25 | *N_e_* = 5 | | *N_e_* = 10 | *N_e_* = 20 | *N_e_* = 25 |
| 1 SNP/cM | 63* | 57* | 44* | 26 | 51* | 44* | 31* | 18* | 47* | | 41* | 28* | 16* |
| 5 SNPs/cM | 123 | 126 | 135 | 145 | 110 | 112 | 114 | 116 | 104 | | 106 | 107 | 107 |
| 10 SNPs/cM | 131* | 136* | 155* | 182 | 116 | 119 | 123 | 129 | 111 | | 112 | 115 | 117 |
| Family size = 160 | *N_R_* (1, 6, 12) | | | | | | | | | | | | |
|  | 1 | | | | 6 | | | | | 12 | | | |
|  | *N_e_* = 5 | *N_e_* = 10 | *N_e_* = 20 | *N_e_* = 25 | *N_e_* = 5 | *N_e_* = 10 | *N_e_* = 20 | *N_e_* = 25 | *N_e_* = 5 | | *N_e_* = 10 | *N_e_* = 20 | *N_e_* = 25 |
| 1 SNP/cM | 63* | 56* | 40* | 19* | 46* | 39* | 25* | 10* | 42* | | 35* | 21* | 9* |
| 5 SNPs/cM | 135* | 140* | 156* | 176* | 114 | 116 | 120 | 124 | 108 | | 109 | 111 | 113 |
| 10 SNPs/cM | 139* | 144* | 161* | 185* | 114 | 119 | 123 | 128 | 111 | | 111 | 114 | 117 |
